# Children’s Emotions after Exposure to News: Investigating Chat Conversations with Peers as a Coping Strategy

**DOI:** 10.1007/s10964-021-01408-0

**Published:** 2021-02-20

**Authors:** Ming Ebbinkhuijsen, Kirsten E. Bevelander, Moniek Buijzen, Mariska Kleemans

**Affiliations:** 1 Behavioural Science Institute, Radboud University, Nijmegen, The Netherlands; 2 Radboud Institute for Health Sciences, Primary and Community Care, Radboud University and Medical Center, Nijmegen, The Netherlands; 3 Erasmus School of Social and Behavioural Sciences, Erasmus University Rotterdam, Rotterdam, The Netherlands

**Keywords:** Children, News, Emotions, Coping, Chat conversations

## Abstract

Hardly any research has been conducted regarding coping strategies that children can use in response to negative news, although they are frequently exposed to and emotionally affected by such news. Chat conversations with peers about the news could be a coping strategy for children in this regard. To investigate this, children (*N* = 307; 46.3% girls; *M*_age_ = 10.51; *SD*_*age*_ = 0.98; range 8–13 years old) participated in a preregistered experiment in which their emotions were measured before and after exposure to a news video on a smartphone and also after a postexposure activity (i.e., chatting about the news as an experimental condition versus chatting about something else or solving a puzzle as control conditions). The results showed that the decrease in negative emotions and the increase in positive emotions were weaker for children who chatted about the news than for those in the control conditions. Thus, seeking social support in online chat conversations did not have the anticipated effect—and might even have an adverse effect.

## Introduction

Many children of primary school age experience negative emotions after watching television news (Riddle et al. 2012). Watching the news with parents or under the supervision of a teacher might help children to cope with negative emotions, for instance, because adults can provide explanations or can reassure children (Walma van der Molen 2004). However, because today’s children more often watch the news alone in an online setting (cf., Apestaartjaren 2020), it is crucial to give them their own tools to handle emotions elicited, particularly by negative news content. As yet, hardly any research has been conducted regarding coping strategies that children can use in this regard. One study showed that real-life conversations with peers about news helped children to cope with their emotions after watching negative news (Kleemans et al. 2017b), but the possibilities that the digital landscape offers in this regard are unexplored. The current study experimentally investigated whether facilitating online chat conversations with peers about negative news helps children to cope with their emotions elicited by this news. The study focused on children between 8 and 13 years old because insights from developmental psychology indicate that children in this age category meet the conditions necessary to understand news—such as being able to distinguish between fiction and nonfiction and having the ability to empathize with others—but also that they are, as a consequence, emotionally sensitive to negative news (cf., Valkenburg and Piotrowksi 2017).

### Children and the News

Exposure to news is deemed important to prepare children for their role as citizens in society (Alon-Tirosh and Lemish 2014). Because adult news is cognitively and emotionally difficult to follow for children, news specifically targeting children—mainly in the form of television news—was introduced in several Western countries since the 1970s. The format of children’s television news programs differs to a certain extent across countries, but in most countries—including the Netherlands, where the current study was conducted—it is comparable to the adult news format. For instance, there is a daily newscast presented by an (adult) news anchor in the studio, covering several current events happening in the world—such as news about politics, natural disasters, accidents and culture. Events are presented in longer and shorter news stories, with different types of sources (e.g., eyewitnesses, experts, vox pops) who comment on the event. However, children’s news deviates in other aspects from the adult news because the producers try to tailor it to children’s needs. For example, there is more attention given to positive or humorous news, to stories with relevance to children’s lives, and the stories are presented in an understandable language (Walma van der Molen and de Vries 2003). In the Netherlands, a daily morning bulletin is broadcast during school hours, while a longer newscast is aired after dinner time to watch at home. The newscasts and specific videos are also provided via the website and social media of the public service news organization. The audience ratings for both television and online are consistently high and are increasing particularly for online content (NOS 2019).

The rise of children’s news has led to increased scholarly attention to the topic over the past decades. An important line of research has been investigating the effects that exposure to news may have on children. In most instances, these studies have focused on negative effects by showing that children display increased negative emotions (e.g., Riddle et al. 2012; Walma Van Der Molen et al. 2002), decreased positive emotions (Kleemans et al. 2017a) or even behaviors such as having nightmares, stomach aches, crying and rumination after news exposure (e.g., Smith and Moyer-Gusé 2006). Following this line of thought, attention is raised to the question how children’s news can be better tailored to children’s emotional needs (e.g., Kleemans et al. 2017b). Another line of studies discusses news for children from the perspective that children have civic rights in democratic societies and thus need to be adequately informed. In these studies, it is questioned whether it is necessary and desirable to censor news for children (cf. Carter 2017). Although sometimes presented as two opposite views on children and the news, both lines share the basic idea that it is important to get and keep children involved in the news. It thus seems to be the challenge to find a way to inform children as much as possible about important events while at the same time avoiding that the negative nature of news stories scares children and consequently might lead to news avoidance. Encouraging the use of coping strategies may be helpful here.

### Coping Strategies

Coping is typically referred to as the cognitive and behavioral efforts to deal with, reduce or tolerate consequences of stressful situations (Folkman 1984). Exposure to negative news content can be seen as an example of such a stressful situation. Two types of coping are discerned in this regard. First, proactive coping refers to efforts that are undertaken in advance of a potentially stressful event to prevent or modify it before it occurs (Aspinwall and Taylor 1997, p. 417). With regard to news, an important proactive coping strategy involves news literacy education—that is, empowering people to access, evaluate, analyze and create news (Ashley et al. 2013). For instance, children with higher news literacy levels should be able to find news content that is more suitable for them. Second, reactive coping refers to efforts to deal with a stressful event that is happening at that moment or has already occurred (Schwarzer and Taubert 2002). Previous research on reactive coping strategies distinguishes two main categories of coping: problem- versus emotion-focused coping (Folkman and Lazarus 1980).

These main categories of reactive coping are broad and do not specifically concern children’s coping strategies. For children—explicitly investigated among third through eighth grade children (Lohaus et al. 2018)—examples of reactive coping strategies they especially use are seeking social support, problem-solving, avoidant coping, palliative emotion regulation, and anger-related emotion regulation (externalizing feelings of anger and fury). Problem-solving is the most-often-used coping strategy for both interpersonal and academic stressors by children (Eschenbeck et al. 2007). Examples of this are having an argument with a friend—an interpersonal stressor—or having problems with homework or receiving a bad grade as academic stressors. Problem-solving in this regard refers to, for example, trying to think of different ways to solve the problem (Lohaus et al. 2018). Other often-used strategies, defined as emotion-focused coping, involve palliative emotion regulation—such as relaxation or distraction—and seeking social support (Eschenbeck et al. 2007).

When it comes to dealing with emotions elicited by negative news content (e.g., news about violence, war, disasters, accidents), problem-focused coping is suggested to be ineffective because children cannot exert influence on the source of their experienced emotions (e.g., changing the negative event presented in the news). In addition, relaxation or distraction are more indirect strategies of dealing with a negative event (Eschenbeck et al. 2007), while this study aims to put children into direct action. To empower children to deal with their emotions in a direct way, social support seeking might be an effective tool. Therefore, this study investigated whether social support seeking can be used as a coping strategy to deal with negative news events.

### Seeking Social Support Online

Seeking social support is a typical example of reactive coping (Carver et al. 1989). From infancy, children are already aware that they can turn to their parents or other trusted relatives when experiencing negative emotions (Thompson 1994). In addition, contacting friends was found to be a commonly used and effective reactive coping strategy among children when dealing with certain stressors (Leung 2006). Seeking help from adults and friends was equally likely to happen among children between 7 and 11 years old (Pateraki and Houndoumadi 2001). The literature suggests that peers are sought out for emotional support, indicating that enhancing access to such coping resources can contribute to children’s coping with emotions (Crystal et al. 2008). In addition, research has shown that children’s peers were the most salient providers of emotional processing coping assistance regarding Hurricane Andrew (Prinstein et al. 1996). Also, children who were exposed to television news that emphasized problems and included negative emotions had less negative and more positive levels of emotions after discussing this news with peers in a classroom setting than children who did not participate in peer discussions (Kleemans et al. 2017b). These findings suggest that real-life conversations with peers about news may help children to better cope with their emotions.

Because the internet offers increasing possibilities for children to watch news when they are alone, it is relevant to investigate whether it is also helpful to encourage social support seeking in an online setting. Digital technologies can be used to create such structures of social support. This study investigated one of these digital opportunities, namely facilitating online chat conversations about negative news. The focus in the current study is on conversations with peers because peers and the corresponding social interactions between them become increasingly important as children get older and become adolescents. To be more specific, children become highly committed to the peer group to which they belong (cf., Valkenburg and Piotrowski 2017), and it can thus be expected that the social support of peers can be effective in this phase of life (Kleemans et al. 2017b).

Children between approximately 8 and 13 years of age were central to this study because they are in the developmental stage in which this issue is most relevant. Children from the age of 8 and above become cognitively mature enough to follow the news. In particular, they are able to understand that news is real, have increased interest in real-world phenomena and can empathize with others (cf. Valkenburg and Piotrowski 2017). On a socioemotional level, this leads to the children becoming more vulnerable (i.e., experiencing fierce negative emotions) to negative content in the news because they understand the impact of negative events (Smith and Wilson 2000). Thus, it is particularly important for this age group to investigate whether seeking social support from peers online is helpful here.

The expectation is that after participation in an online chat conversation with peers about the news they have watched, these children’s emotions would recover more strongly compared to the emotions of those who did not participate in an online chat conversation with peers about the news. The focus in this regard is on both negative and positive emotions because past research has shown that negative news not only increases negative feelings such as sadness and anxiety but also decreases positive feelings such as joy and happiness (e.g., Kleemans et al. 2017b). In addition, potential individual differences in reactive coping are considered. No specific expectations could be distinguished in this regard. It might be that children have a preferred coping strategy they use across different situations, that they have specific personality variables that affect coping strategy preferences or that preferred coping strategies exert an influence on specific responses (Carver et al. 1989). There does not seem to be conformity with respect to which strategy seems to be most preferred in a given situation (Eschenbeck et al. 2007; Hampel and Petermann 2005), but research suggests that individual differences in the preference for particular coping strategies might influence children’s use of coping strategies during specific situations (Ayers et al. 1996). Therefore, exploring whether a certain preference for social support seeking as a reactive coping strategy would affect the influence of online chat conversations with peers on children’s emotions is necessary.

## Current Study

Negative news affects children’s negative and positive emotions; therefore, it is important to give them tools to help them cope with these emotions. Given the lack of knowledge on children’s coping strategies with regard to negative news exposure, this study experimentally investigated whether seeking social support would be an effective coping strategy in this regard. The focus is on facilitating online chat conversations about news with a small group of (anonymous) peers from their own class as strategy to cope with emotions elicited by negative news. Coping strategies in general (Eschenbeck et al. 2007) and specifically social support seeking (Kleemans et al. 2017b) have been shown to decrease the negative effect of stressors on children’s emotions. Thus, in the current study, the expectation is that after participation in an online chat conversation with peers about news to which they were exposed, children show (a) a stronger decrease in negative emotional responses and (b) a stronger increase in positive emotional responses than children who did not participate in an online chat conversation with peers about the news (Hypothesis 1). Moreover, it is questioned whether children who are generally more likely to seek social support after exposure to negative news benefit more or less from online chat conversations with peers than children who are generally less likely to use seeking social support as a coping strategy (Research question 1).

## Methods

A preregistered experiment (on the Open Science Framework website) was conducted in classrooms using mobile phones. Children were exposed to a news video and engaged in an activity thereafter. Their emotional responses were measured at three time points: T1 = before news exposure, T2 = directly after news exposure, and T3 = after the postexposure activity. The activity manipulation to test social support seeking as a coping strategy included either online chatting about news (experimental condition), online chatting about another topic (active control condition) or solving a puzzle (passive control condition). To account for potential variations in different news stories, five different videos were used as stimulus materials. The moderator variable social support seeking likelihood was measured in a questionnaire at T1. Thus, the repeated-measures mixed design included one within-subjects factor (time), two between-subjects factors (postexposure activity, story topic) and a moderating variable (social support seeking likelihood). The study received the approval of the ethics committee of the host university.

### Participants

Based on an a priori power analysis using G*Power (Faul et al. 2007), the aim was to recruit a sample size of *N* = 300 to detect small effects (*α* = 0.05; effect size = 0.14; 95% power). Children in grades 4–6 were recruited at five primary schools in the Netherlands. After obtaining the consent of each school’s principal, parents received an information letter about the study, stating that the study focused on responses to negative news content and that children would be exposed to a news story that was previously broadcast on television. It also included a request for passive consent. Four parents did not allow their child to participate. Before the start of the experiment, children were informed about the study, using a cover story about the aim of the study. Children were told that the producers of the Dutch children’s news wanted to hear their opinion on it and thus that they would be exposed to one news video and would then be asked to answer questions regarding that story. In addition, they were asked to assent to participation. One child did not want to participate.

In total, 326 children took part in the experiment. However, due to technical problems with the mobile phones and because some children did not follow the instructions (e.g., they could not watch the news video or filled out the questions about the postexposure activity before participating in this activity), 19 participants were removed from the dataset. Therefore, the final sample consisted of 307 children (53.7% boys; *M*_*age*_ = 10.51; *SD*_*age*_ = 0.98; range 8–13 years old) from grade 4 (*N* = 98), grade 5 (*N* = 97) and grade 6 (*N* = 112). Most of them (85.0%) indicated that they watched the Dutch children’s news at least once a week, with a mean of 3.24 days a week (*SD* = 2.18).

### Stimulus Materials

To test the effect of chat conversations as a coping strategy, emotions should be triggered in children. To this end, five news videos were used—all previously presented on the website of the Dutch children’s television news program NOS Jeugdjournaal. To select these videos, a pretest in which nine videos were tested (six negative, three neutral) was conducted.

#### Pretest

A total of 89 children (42.7% boys; *M*_*age*_ = 10.17; *SD*_*age*_ = 0.94) from two schools (grade 4–6) that were not included in the main study participated in the pretest. The passive consent of their parents was sought. Three children did not want to participate; the other participants signed the assent form. After being informed about the study, they each received a smartphone on which they first had to answer some general questions about their age, sex and grade. Next, they watched five out of nine randomly selected videos on the smartphone.

Three videos reported on a natural disaster (1 = a hurricane in the Philippines, 2 = a volcanic eruption in Japan and 3 = an earthquake in Nepal), three on an accident (4 = a plane crash in France, 5 = an explosion in China and 6 = a train accident in Belgium), and three videos on neutral stories (7 = an important ice-skating match for children, 8 = the busiest day at Schiphol Airport and 9 = double surnames). To increase comparability between the videos, some storylines were edited slightly to make sure that all videos had the same structure and that they all lasted between 110 and 112 s.

After watching each video, participants filled out a short questionnaire. Their emotional responses (i.e., fear, sadness, happiness and enthusiasm) were measured using Visual Analog Scales (VAS) ranging from 0 to 100. Additionally, children had to evaluate each news video in general on a scale from 1 (being very bad) to 10 (very good). Thereafter, they reported how positive or negative they perceived the video to be and how terrible and important they perceived the event to be, using scales all ranging from 1 (not at all) to 10 (very much). Because the main study aimed to measure children’s responses to negative news videos, similar scores on the negative emotional responses (fear and sadness) and positive emotional responses (happiness, enthusiasm) were the most important criteria in the selection of the five videos. The other measurements were used as an additional check to be sure that the videos did not differ much with respect to the other criteria. The results of the pretest are summarized in Table 1.

**Table 1:** Means scores and standard deviations for each variable broken down for the nine videos

	Natural disaster			Accident			Neutral		
	1	2	3	4	5	6	7	8	9
Fear	28.85 (29.45)	37.93 (28.74)	36.65 (29.36)	38.33 (30.07)	35.31 (30.93)	33.93 (29.65)	4.00 (10.40)	4.36 (11.75)	5.95 (16.23)
Sadness	39.00 (33.65)	45.90 (29.88)	44.55 (29.72)	48.74 (34.10)	44.51 (30.81)	38.33 (30.87)	3.44 (9.09)	7.18 (19.24)	6.33 (14.00)
Happiness	42.02 (34.03)	36.00 (25.81)	35.10 (29.63)	40.10 (34.97)	35.87 (30.58)	41.10 (29.94)	69.38 (25.23)	67.97 (30.19)	70.51 (27.90)
Enthusiasm	38.17 (30.58)	33.28 (25.90)	32.30 (24.11)	39.54 (32.43)	34.82 (28.07)	32.08 (24.02)	58.77 (33.03)	52.92 (32.13)	57.54 (34.08)
Evaluation	7.83 (1.58)	7.61 (1.93)	7.93 (1.77)	7.69 (1.78)	8.23 (1.65)	8.25 (1.35)	7.85 (1.50)	7.90 (1.80)	7.38 (2.44)
Positivity	5.00 (2.85)	5.03 (2.65)	4.93 (2.69)	4.46 (3.07)	4.33 (2.39)	5.40 (2.68)	7.82 (1.78)	7.31 (2.28)	7.03 (2.65)
Negativity	5.44 (2.75)	5.88 (2.31)	6.20 (2.63)	6.03 (2.68)	5.49 (2.89)	5.55 (2.43)	2.15 (1.81)	2.87 (2.17)	3.95 (2.93)
Terribleness	8.46 (1.50)	7.85 (1.99)	9.00 (1.43)	8.49 (1.65)	8.72 (1.67)	7.80 (1.81)	2.87 (2.34)	3.08 (2.22)	3.79 (2.99)
Importance	8.05 (1.79)	7.75 (1.78)	8.55 (1.30)	8.00 (1.73)	8.38 (1.70)	8.05 (1.43)	6.64 (2.40)	6.38 (2.29)	6.97 (2.76)

1 = a hurricane in the Philippines, 2 = a volcanic eruption in Japan, 3 = an earthquake in Nepal, 4 = a plane crash in France, 5 = an explosion in China, 6 = a train accident in Belgium, 7 = an important ice-skating match for children, 8 = the busiest day at Schiphol Airport, 9 = double surnames

#### Main study materials

For the main study, the four negative videos that were most comparable in mean scores for each variable (i.e., the volcanic eruption in Japan (2) and the earthquake in Nepal (3) for natural disasters; the plane crash (4) and the explosion in China (5) for accidents) were selected. In addition, video 8 (about the busiest day at Schiphol Airport) was selected as the control condition because it produced the most neutral feelings. This neutral control condition was included to check the assumption that it was the negativity in the story that elicited the expected increase in negative and decrease in positive emotions.

### Main Study Measures

#### Negative emotional responses

To measure negative emotional responses, children indicated at T1–T3 how scared and sad they felt using two visual analog scales (VAS) ranging from 0 to 100 (Kleemans et al. 2017a). Because of significant correlations between the two negative emotions at T1 (*r* = 0.241; *p* < 0.001), T2 (*r* = 0.609; *p* < 0.001), and T3 (*r* = 0.611; *p* < 0.001), a mean score was calculated for negative emotions at each time point (see Table 2).

**Table 2:** Descriptive statistics for the dependent variables at three time points

	Negative emotions	Positive emotions
T1 (Before news exposure)	6.79 (13.46)	72.97 (20.11)
T2 (After news exposure)	18.78 (22.90)	54.15 (27.79)
T3 (After activity)	10.68 (18.78)	69.04 (25.63)

*N* = 307 (entire sample)

#### Positive emotional responses

To measure positive emotional responses, children indicated at T1–T3 how happy and enthusiastic they felt using a VAS ranging from 0 to 100 (Kleemans et al. 2017a). A mean score was calculated for positive emotions at each time point (see Table 2) due to the significant correlations between the two emotions at T1 (*r* = 0.517; *p* < 0.001), T2 (*r* = 0.687; *p* < 0.001) and T3 (*r* = 0.670; *p* < 0.001).

#### Social support seeking likelihood

To measure the moderating variable social support seeking likelihood, a slightly adapted version of the Seeking Social Support Strategy Scale of Causey and Dubow (1992) was used. This scale consists of eight items, which were all included in the questionnaire at T1. Responses were measured using a 6-point scale ranging from “never” (1) to “always” (6). The starting question for each item was adapted to the news context of the study: “When I see a story on the news about a dramatic event, one that is more dramatic than I normally see, I usually…”, followed by the eight items formulated by Causey and Dubow (1992), such as “… talk to somebody about how it made me feel” and “… get help from a family member”.

A principal factor analysis was conducted for the eight items. The criterion of factors with eigenvalues >1 yielded one component (EV = 4.627; 57.83% variance explained). Cronbach’s alpha of the scale was high (*α* = 0.894). Thus, a mean score was calculated based on the eight items to indicate children’s social support seeking likelihood (*M* = 2.63; *SD* = 1.09). Then, this variable was dichotomized using a mean split to make it suitable for the analyses. As a result, 170 children were assigned to the “lower social support seeking” group and 137 to the “higher social support seeking” group.

#### Covariates

Besides sex, age and grade, children were asked at T1 whether they possessed a smartphone (74.9% said “yes”), how often they typed messages on a smartphone on a scale ranging from “never” (1) to “very often” (6) (*M* = 3.45; *SD* = 1.54) and how often they watched children’s TV news on a scale ranging from 0 to 7 days a week (*M* = 3.24; *SD* = 2.18). At T3, they were asked how much they enjoyed the postexposure activity with a 10-point VAS (*M* = 7.70; *SD* = 2.85).

### Procedure

Children from the same class participated in the experiment at the same time in their own classroom. Each child was randomly handed a mobile phone on which one of the five news stories was pre-installed (number of participants per video ranged between *N* = 59 and *N* = 62). After children had answered several questions (e.g., about demographics, smartphone use, social support seeking likelihood) on their phone, they indicated how they felt at that moment. Then, the news video was shown, followed by the second measurement about how they felt (T2). Finally, they took part in one of the three postexposure activities, which each lasted for five minutes. Two groups were handed another mobile phone to chat with peers about the news (*N* = 101) or about something else (*N* = 95), while the third group solved a word search puzzle on paper (*N* = 111). A word search puzzle was chosen to keep these children occupied for the same amount of time as the other two conditions. Due to technical inabilities, it was not possible to run the app for the chat conversation on the same mobile phone as the questionnaire. Randomization checks showed that participants were equally divided over the three conditions, based on sex, χ^2^(2) = 1.672, *p* = 0.433, age, *F*(5, 297) = 1.037, *p* = 0.396 and class level, *F*(2, 297) = 0.679; *p* = 0.508.

For both chat conditions, children participated in a chat group consisting of four to five classmates (each on their own device), who had all watched the same news video. They were instructed not to reveal their identity, but they were aware that they were chatting with peers from their own class. It was decided to make the conversations anonymous—so they would not know with whom they were in the same chat group—in order to avoid that the children would also start verbal or nonverbal communication during the experiment with the other children in the same chat conversation. Despite being in the same classroom, children took this very seriously and did not send any (non)verbal cues revealing which group they were in while participating in this activity.

Researchers stimulated and guided the chat conversations by posting three questions. Questions for the experimental condition were about what came to mind when thinking about the news they had just seen and which answers of other children for this question they found important. In addition, children were asked to decide together what the three most important things were that they remembered. In the active control condition, participants received three questions unrelated to the news video (how often they visit the various media channels the children’s news uses to inform children, how often they follow news other than the Dutch children’s news, and what medium they use most to follow the news). When children only answered the questions with “yes” or “no”, researchers asked why. The researchers did not moderate the conversations any further than that. These chat conversations lasted for five minutes.

After the researcher marked the end of the activities, children completed the last set of questions about how they felt (T3). Additionally, they indicated how much they liked the postexposure activity. Afterwards, they were debriefed about the real aim of the study and thanked for their participation. They had the opportunity to ask questions or share concerns.

### Analysis Procedure

Before conducting the analyses, which covariates were necessary to include was investigated by calculating correlations between the potential covariates (sex, age, grade and television news viewing behavior) and the dependent variables at the three time points. At T3, the smartphone use variables and children’s enjoyment of the postexposure activity were included (see Table 3). Only a consistent, significant correlation between sex and negative emotional responses was found. Therefore, sex was included as the covariate when analyzing effects on negative emotions (i.e., in the preliminary and main analyses). Furthermore, there was a significant correlation between positive emotional responses and the enjoyment of the postexposure activity at T3. Therefore, this variable was included in the main analysis for positive emotions.

**Table 3:** Correlation table for potential covariates (related to the dependent variables)

	Negative emotions			Positive emotions		
	T1	T2	T3	T1	T2	T3
Age	−0.011	−0.124^*^	−0.084	−0.109	0.049	0.043
Sex	0.227**	0.209**	0.163**	0.024	−0.048	−0.057
Grade	−0.049	−0.133*	−0.099	−0.116*	0.044	0.013
Children’s television news viewing behavior	0.070	0.098	0.067	−0.053	−0.148**	−0.073
Possession of smartphone	–	–	0.022	–	–	0.087
Typing on smartphone	–	–	−0.055	–	–	−0.045
Enjoyment of postexposure activity	–	–	0.029	–	–	0.332**

*p < 0.05, **p < 0.001, *N* = 307 (entire sample)

As the preliminary analysis, the assumption underlying this study that exposure to negative news leads to increased negative emotional responses and decreased positive emotional responses was tested. This condition had to be met in order to conduct and interpret the main analyses.^1^ Repeated measures ANCOVAs were conducted for negative and positive responses. The emotions measured before (T1) and directly after (T2) exposure as within-subject factors were included. Story topic was included as a between-subjects factor to investigate whether the four negative videos had comparable effects on emotions and whether they differed from the neutral video. Moreover, the content of the chat conversations is presented as the preliminary analysis because it is important to have a sense of the actual content of the discussion for the interpretation of the results.

For the main analysis, the full model was tested for negative emotions and positive emotions separately, using ANCOVAs, including time as a within-subjects variable (emotions at T1, T2, T3) and postexposure activity, social support seeking likelihood and story topic as between-subjects variables. Children exposed to the neutral video were excluded because the effects of social support seeking on emotions were only relevant to investigate for children exposed to negative news (*N* = 245). Social support seeking likelihood was included as a moderating variable in the main analysis and interpreted based on the change in negative and positive emotions between T2 and T3. In both analyses, Mauchly’s test indicated that the assumption of sphericity had been violated, based on the ε > 0.75 rule (Field 2013). Therefore, the Huynh-Feldt correction was used to analyze the within-subject effects. Post hoc *F*-tests with Bonferroni corrections were used to compare the between-groups effects.

## Results

### Preliminary Analysis: Effects of News on Emotions

As a prerequisite for testing potential effects of chat conversations as a coping strategy, it had to be certain that the negative videos used in the experiment would indeed elicit emotions. For negative emotions, the analysis yielded a marginally significant effect of time (*p* = 0.078) and a significant effect of story topic, *F*(4, 301) = 4.351; *p* = 0.002; *η*^*2*^_p_ = 0.06, further qualified by the significant interaction between time and story topic, *F*(4, 301) = 9.248; *p* < 0.001; *η*^*2*^_p_ = 0.11. Post hoc analyses showed that negative emotions significantly increased between T1 and T2 for all four negative videos (*p* < 0.001 each), whereas the negative emotions did not change for the neutral video (*p* = 0.519); see Table 2. This confirms that the negative videos had the assumed effect. Children’s negative emotions not only increased after exposure to negative news but also became more negative than the emotions from children who watched the neutral video. The effect of the covariate was also significant, *F*(1, 301) = 21.321; *p* < 0.001; *η*^*2*^_p_ = 0.07. Girls reported higher levels of negative emotions than boys.

For positive emotions, findings showed a main effect of time, *F*(1, 302) = 155.661; *p* < 0.001; *η*^*2*^_p_ = 0.34, but no main effect of story topic (*p* = 0.230). However, the interaction between time and story topic was significant, *F*(4, 302) = 7.242; *p* < 0.001; *η*^*2*^_p_ = 0.09. As assumed, each negative video caused a decrease in positive emotions between T1 and T2 (*p* < 0.001 each), whereas positive emotions remained stable for the neutral video (*p* = 0.378); see Table 4. Thus, the negative videos also affected the positive emotions as intended.

**Table 4:** Mean scores and standard errors for the dependent variables per story topic at T1 and T2

	Negative emotions		Positive emotions	
	T1	T2	T1	T2
Volcanic eruption in Japan	9.16 (1.71)	23.67 (2.80)	71.55 (2.61)	51.23 (3.55)
Earthquake in Nepal	4.46 (1.65)	20.36 (2.71)	72.71 (2.53)	49.84 (3.43)
Plane crash in France	7.31 (1.68)	25.48 (2.76)	74.12 (2.57)	48.80 (3.45)
Explosion in China	5.36 (1.67)	18.60 (2.74)	77.38 (2.55)	54.68 (3.46)
Busiest day at Schiphol Airport	7.80 (1.67)	6.13 (2.73)	69.03 (2.55)	66.07 (3.46)

Differences between T1 and T2 were significant (*p* < 0.001) for all videos, except for the video about the busiest day at Schiphol Airport (*p* = 0.519 for negative emotions; *p* = 0.378 for positive emotions), *N* = 307 (entire sample)

### Preliminary Analysis: Content of the Chat Conversations

To gain insights into the content of the chat conversations, the transcriptions of what children typed in the chat was explored. This showed that the quality of the social supportiveness of the conversations was comparable between all chat conversations. In each chat group discussing the news they had just seen (experimental condition), children talked about what they had remembered most from the video, and they expressed their emotions in words or by using emoticons. For example, one child reported: “There was a plane crash, and people were very sad about it”. When the researchers asked which answers from other children they found important, they generally agreed with each other. For instance, one child reported: “I also feel bad for them”. After responding to each other’s answers, they together chose the three most important things they remembered from the news video. Overall, the chats in the experimental condition showed that the children adhered to the rules presented to them. There were no indications that the children talked about other things than those requested. The chat conversations in the active control condition did not include mentions of the news they had just seen. In this condition, children answered the general questions about news consumption as asked by the researchers.

### Main Analysis: Effects of Coping Strategies on Emotions

**Fig. 1:**
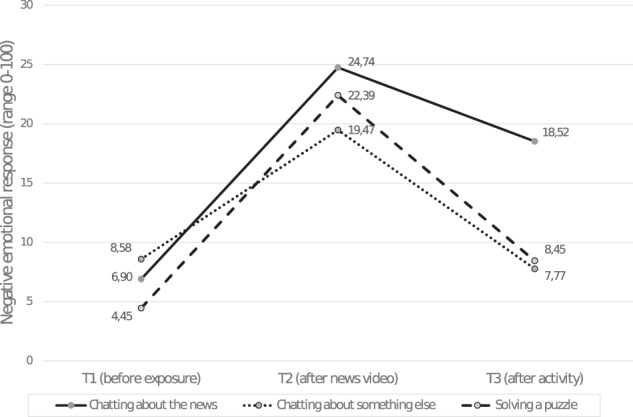
Negative emotions at three time points for each of the three activities separately. This figure represents the sub-portion of the sample that was only exposed to negative news content (*N* = 245)

First, the change in negative emotional responses before (T2) and after (T3) partaking in the postexposure activity (i.e., news-related chat conversation, nonrelated chat conversation or solving a puzzle) was analyzed. A stronger decrease in negative emotional responses for children participating in an online chat conversation about the news than for children in the other two conditions was predicted. In the analyses, only marginal significant main effects of postexposure activity (*p* = 0.078) and time (*p* = 0.055) were found. Moreover, the interaction between time and postexposure activity was significant, *F*(3.863, 424.915) = 4.955; *p* = 0.001; *η*^*2*^_p_ = 0.04. As illustrated in Fig. 1, children who chatted about the news showed a smaller decrease in negative emotional responses (*M*_*ΔT2–T3*_ = −6.22; *SE* = 2.02) than children who chatted about something else (*M*_*ΔT2–T3*_ = −11.70; *SE* = 2.14) or solved a puzzle (*M*_*ΔT2–T3*_ = −13.94; *SE* = 1.86).

Posthoc tests showed that the differences in negative emotions were not significant at T1 (*p* = 0.122) and at T2 (*p* = 0.415). However, at T3 they differed significantly, *F*(2, 220) = 8.103; *p* < 0.001; *η*^*2*^_p_ = 0.07, with children chatting about the news reporting a significantly higher level of negative emotions than children chatting about something else (*p* < 0.001) or solving a puzzle (*p* < 0.001). The difference between the latter two conditions was not significant (*p* = 0.818). Moreover, for children in the news-related chat condition, their emotions differed significantly between each combination of time points (*p* < 0.001 each). For the children in the nonrelated chat condition, it was found that the emotions at T1 and T3 were comparable (*p* = 0.679) but differed significantly between T1 and T2, and T2 and T3 (*p* < 0.001 each). For children who solved a puzzle, the differences in negative emotions were the largest between T1–T2 and T2–T3 (*p* < 0.001) but were also significant between T1 and T3 (*p* = 0.021).

No main (*p* = 0.137) or interaction (story topic*postexposure activity: *p* = 0.729) effects for story topic were found, implying that the observed effects were comparable for all four negative videos. The effect of sex was significant, *F*(1, 220) = 10.703; *p* = 0.001; *η*^*2*^_p_ = 0.05, with girls showing higher levels of negative emotions than boys. In sum, results showed that there was a weaker—instead of the predicted stronger—decrease in negative emotions for children participating in an online chat conversation about the news than for children who did another postexposure activity.

**Fig. 2:**
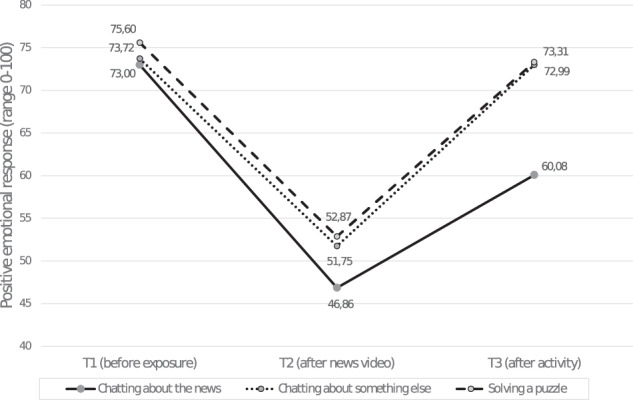
Positive emotions at three time points for each of the three activities separately. This figure represents the sub-portion of the sample that was only exposed to negative news content (*N* = 245)

For positive emotions, a stronger increase between T2 and T3 in positive emotional responses was expected for children participating in an online chat conversation about the news than for the other children. Aside from a main effect of time, *F*(2, 440) = 11.673; *p* < 0.001; *η*^*2*^_p_ = 0.05, and activity, *F*(2, 220) = 3.212; *p* = 0.042; *η*^*2*^_p_ = 0.03, results showed a significant interaction between time and postexposure activity, *F*(4, 440) = 2.753; *p* = 0.028; *η*^*2*^_p_ = 0.02. Figure 2 shows that chatting about the news led to a smaller increase in positive emotions (*M*_*ΔT2–T3*_ = 13.22; *SE* = 2.82) than chatting about something else (*M*_*ΔT2-–T3*_ = 21.24; *SE* = 2.98) or solving a puzzle (*M*_*ΔT2–T3*_ = 20.45; *SE* = 2.66).

Post hoc tests showed that the differences in positive emotions were not significant at T1 (*p* = 0.690) and at T2 (*p* = 0.364). However, at T3 they differed significantly, *F*(2, 220) = 7.420; *p* < 0.001; *η*^*2*^_p_ = 0.06, with children who chatted about the news having a significantly lower level of positive emotions than those who chatted about something else (*p* = 0.001) or solved a puzzle (*p* = 0.001). The difference between the latter two conditions was not significant (*p* = 0.936). In addition, for children in the news-related chat condition, emotions differed significantly between each of the time points (*p* < 0.001 each). For the nonrelated chat condition and the puzzle condition, analyses showed that the emotions at T1 were at a comparable level as at T3 (respectively *p* = 0.787 and *p* = 0.341), but differed significantly between the other time points (*p* < 0.001 each).

The effects were comparable for all negative videos because the main (*p* = 0.475) and interaction (story topic*postexposure activity: *p* = 0.290) effects for story topic were not significant. The effect of the control variable enjoyment of the postexposure activity was significant, *F*(1, 220) = 13.389; *p* < 0.001; *η*^*2*^_p_ = 0.06, showing that children liked the two chat conditions more than solving the puzzle. In sum, the findings showed that there was a weaker—instead of the predicted stronger—increase in positive emotions for children participating in an online chat conversation about the news than for children who participated in another postexposure activity.

### The Role of Social Support Seeking

It was also questioned whether children who are generally more likely to seek social support after exposure to negative news benefited less or more from online chat conversations with peers compared to children who are generally less likely to use seeking social support as coping strategy. To investigate this, the three-way interaction between time, postexposure activity and social support seeking likelihood was of key interest. This interaction showed whether the effect of the postexposure activity on the change in emotions between T2 and T3 was moderated by social support seeking likelihood.

For negative emotional responses, no significant interaction of time, postexposure activity and social support seeking likelihood was found (*p* = 0.554). However, results yielded a main effect for social support seeking likelihood, *F*(1, 220) = 3.955; *p* = 0.048; *η*^*2*^_p_ = 0.02, showing that children with higher levels of social support seeking likelihood had somewhat higher levels of negative emotional responses (*M* = 15.51; *SE* = 1.51) than children with lower levels of social support seeking likelihood (*M* = 11.45; *SE* = 1.36). For positive emotional responses, no significant three-way interaction of time, postexposure activity and social support seeking likelihood was found (*p* = 0.792), nor a main effect of social support seeking likelihood (*p* = 0.471). Taken together, there is no reason to assume that differences in social support seeking likelihood affected the effects of the postexposure activities on children’s emotions.

## Discussion

Although there is ample evidence that children are emotionally affected by children’s news, hardly any research pays attention to strategies that can help children to cope with their emotions after exposure to negative news. Therefore, this study investigated whether seeking social support from peers could function as an effective coping strategy in this regard. Particular focus was on seeking support from peers in an online setting because children increasingly access news online without adult supervision. This study investigated whether facilitating online chat conversations about negative news with peers supported children (8–13 years old) in coping with their emotions elicited by this news. Children reported higher levels of negative and lower levels of positive emotions after online chat conversations about the news than children who participated in one of the control conditions. When children participated in a nonrelated chat conversation or solved a puzzle, their levels of negative and positive emotions were (almost) similar at the end as at the beginning of the experiment (i.e., before news exposure). For children in the news-related chat conversation, their level of negative emotions remained higher and the level of positive emotions lower than before the news exposure. This suggests that seeking social support online does not have the anticipated coping effect.

There are two ways to interpret the observed patterns. On the one hand, the findings may suggest that all postexposure activities were useful to help children to cope with their emotions (see difference between T2 and T3) but that distracting children’s attention from negative news (i.e., control conditions) was more effective than seeking the support of peers (i.e., experimental condition). This would imply that the activities in the active and passive control condition were coping strategies in their own right, which is in line with previous research which indicates that distraction is an effective coping strategy for dealing with stressors (Eschenbeck et al. 2007). In addition, both chatting via a mobile phone and solving a puzzle can be seen as a form of play for children, which has been argued to reduce children’s emotions in stressful situations (Capurso and Pazzagli 2016). The findings in the current study support the necessity to investigate distraction and play as potential reactive coping strategies in future research.

On the other hand, it is conceivable that the experimental postexposure activity had an opposite effect and that the control activities had no effect. Specifically, chatting about the news may have partly maintained the children’s emotional state because it stimulated them to keep ruminating on the negative news. In contrast, the emotions of the children in the control conditions returned to the original level, which might have reflected the natural process that emotions are generally most intense directly after exposure and automatically start to decrease thereafter. This would imply that the investigated coping strategy was not only less effective than the control activities but that it was even harmful by interfering with the natural recovery from fierce emotions over time. Therefore, future research should add a control condition without a postexposure activity to isolate the time effect. Additionally, the effects of all conditions were only examined directly after the five minutes spent on the postexposure activity. It would also be interesting to investigate what happens when children have more time to participate in the postexposure activities and what more long-term influences on their emotions are.

The current study found no indications that differences in social support seeking likelihood moderated the effects of the postexposure activities on children’s emotions. Previous research focusing on the influence of cognitive development and gender on coping showed that there are differences in preferences for coping strategies between older and younger children (Baráth 2002) and between boys and girls (Eschenbeck et al. 2007). However, differences in preferred coping strategies do not necessarily entail that other coping strategies are less or even not effective, as the current study has shown. This issue deserves more attention in future research because it might be beneficial to teach children particular coping strategies that are more effective than their most preferred ones.

The age of the participants may have played a role in the study findings. In particular, the oldest children in this study were on the threshold of adolescence (cf., Valkenburg and Piotrowski 2017). With regard to emotions, this developmental phase is characterized by fast and fundamental biological, cognitive, social and emotional alterations (Theurel and Gentaz 2018). The effect of coping strategies may significantly shift within these years, and social support seeking might become particularly more effective for older age groups. Related to this, differences in children’s digital skills might have played a role (Botha and Ford 2008). Especially younger children in the sample had more difficulty with participating in chat conversations via a mobile phone (e.g., they had problems typing a message or following the conversation because they were slow readers), which may have affected the results to a certain extent. Thus, including older participants (i.e., adolescents) is relevant for future research.

Sex was shown to be a consistent covariate in the analysis regarding negative emotional responses and played a role in these findings. As consistently found in previous research (e.g., Kleemans et al. 2017a; Riddle et al. 2012), girls display higher negative emotional responses than boys at each time point. Causey and Dubow (1992) argue that girls are more likely to use problem-solving and support seeking as coping strategies, while boys more often use distancing (which they might have done in their minds by cognitively reframing or ignoring the negative emotional reactions) and externalizing as coping strategies. Therefore, future research should give attention to sex differences in coping strategies.

This study was, of course, not without limitations. A first limitation that deserves attention is that news stories from a children’s news program were used as stimulus materials, whereas adult news content might have an even stronger impact on children’s emotions. For ethical reasons, children were not exposed to such content, but this may have led to an underestimation of the effect of reactive coping. Second, there is no information about the children’s social context and typical social dynamics. This would be highly interesting to explore because there might be personal differences (other than sex and age) that affect the results—such as frequency of phone use, what they use their phone for, the size of children’s social networks and their peer relations. Moreover, the sample in this study was relatively homogenous, and it would be interesting and important to consider potential sociodemographic factors such as socioeconomic status or cultural and ethnic background, as they might be especially relevant as well. By including individual differences, more specific recommendations can be made regarding which coping strategy is most effective and for whom.

Finally, seeking social support is defined as reaching out for (emotional) help to others (Eschenbeck et al. 2007). However, in this experiment, children participated in a chat conversation in which the researchers posted questions that the children discussed (anonymously) with peers whom they could not seek out themselves. This was done for reasons of comparability so that there were no differences regarding the conversations between the chat groups in the same condition. Nonetheless, this might have affected the ecological validity in this regard. This procedure did not exactly mimic an actual conversation because children directly answered the questions of the researcher. In real life, children should have the chance to choose themselves what specific things from the news they would like to talk about with which peers and not anonymously to fully capture the concept of social support seeking.

## Conclusion

Today’s digital news landscape provides both opportunities and risks for children. To handle emotions elicited by news, scientific insights are warranted with respect to how children can be empowered to cope with it. To this end, the current study investigated online chat conversations with peers as a coping strategy. It was found that encouraging children to partake in activities that are not related to the news content to which they were exposed had a more positive influence on their emotions than children who had chat conversations about the news with peers. This implies that seeking peer support online as a coping strategy seems not as promising as expected with regard to news. In the current (news) media landscape, the challenge for the future is to investigate more closely whether and how the opportunities that digital technologies provide can be used to counteract the negative effects that (online) news exposure may have on children’s socioemotional development.

## Footnotes

1 In the preregistration, this assumption was formulated as Hypothesis 1. However, the structure and readability of the manuscript improved by focusing in particular on the main expectation (i.e., the effect of chat conversations on emotions) and by presenting the test of the basic assumption underlying this study (i.e., that negative news triggers emotions) as the preliminary analysis.
